# Surgical treatment of choanal atresia with transnasal endoscopic approach with stentless single side-hinged flap technique: 5 year retrospective analysis^☆^

**DOI:** 10.1016/j.bjorl.2016.03.009

**Published:** 2016-04-22

**Authors:** Carmelo Saraniti, Manuela Santangelo, Pietro Salvago

**Affiliations:** Università degli Studi di Palermo, Dipartimento di Biomedicina Sperimentale e Neuroscienze Cliniche (BioNeC), Sezione di Otorinolaringoiatria, Palermo, Italy

**Keywords:** Choanal atresia, Endoscopic nasal surgery, Re-stenosis, Atresia de coana, Cirurgia nasal endoscópica, Restenose

## Abstract

**Introduction:**

Choanal atresia is a rare congenital malformation of the nasal cavity characterized by the complete obliteration of the posterior choanae. In 67% of cases choanal atresia is unilateral, affecting mainly (71%) the right nasal cavity. In contrast to the unilateral form, bilateral choanal atresia is a life-threatening condition often associated with respiratory distress with feeding and intermittent cyanosis exacerbated by crying. Surgical treatment remains the only therapeutic option.

**Objective:**

To report our experience in the use of a transnasal endoscopic approach with stentless single side-hinged flap technique for the surgical management of choanal atresia.

**Methods:**

A 5 year retrospective analysis of surgical outcomes of 18 patients treated for choanal atresia with a transnasal technique employing a single side-hinged flap without stent placement. All subjects were assessed preoperatively with a nasal endoscopy and a Maxillofacial computed tomography scan.

**Results:**

Ten males and eight females with a mean age at the time of surgery of 20.05 ± 11.32 years, underwent surgery for choanal atresia. Fifteen subjects (83.33%) had a bony while 3 (26.77%) a mixed bony-membranous atretic plate. Two and sixteen cases suffered from bilateral and unilateral choanal atresia respectively. No intra- and/or early postoperative complications were observed. Between 2 and 3 months after surgery two cases (11.11%) of partial restenosis were found. Only one of these presented a relapse of the nasal obstruction and was subsequently successfully repaired with a second endoscopic procedure.

**Conclusion:**

The surgical technique described follows the basic requirements of corrective surgery and allows good visualization, evaluation and treatment of the atretic plate and the posterior third of the septum, in order to create the new choanal opening. We believe that the use of a stent is not necessary, as recommended in case of other surgical techniques involving the use of more mucosal flaps.

## Resumo

**Introdução:**

A atresia de coanas é uma malformação congênita rara da cavidade nasal caracterizada pela obliteração completa da coana posterior. Nos 67% dos casos a atresia coanal é unilateral, acometendo principalmente (71%) a cavidade nasal direita. Diferentemente da forma unilateral, a atresia coanal bilateral é uma condição com risco de vida, frequentemente associada a angústia respiratória com alimentação e cianose intermitente exacerbada pelo choro. O tratamento cirúrgico permanece como a única opção terapêutica.

**Objetivo:**

Relatar a nossa experiência no uso de uma abordagem endoscópica transnasal com a técnica de retalho articulado de um lado só sem colocação de stent para o tratamento cirúrgico da atresia coanal.

**Método:**

Análise retrospectiva de 5 anos dos desfechos cirúrgicos de 18 pacientes tratados para atresia coanal com uma técnica transnasal com um único retalho de articulação lateral, sem colocação de stent. Todos os indivíduos foram avaliados no pré-operatório com uma endoscopia nasal e um exame de tomografia computadorizada maxilofacial.

**Resultados:**

Dez homens e oito mulheres com idade média no momento da cirurgia de 20,05 ± 11,32 anos foram submetidos a cirurgia para atresia de coanas. Quinze pacientes (83,33%) apresentaram placa atrésica óssea, enquanto 3 (26,77%) apresentaram uma placa atrésica ósseo-membranosa mista. Dois e dezesseis casos sofriam de atresia coanal bilateral e unilateral, respectivamente. Não foram observadas complicações intra e/ou pós-operatórias precoces. Entre 2 e 3 meses após a cirurgia dois casos (11,11%) de restenose parcial foram encontrados. Apenas um deles apresentou uma recidiva da obstrução nasal e, portanto, foi reparado com sucesso com um segundo procedimento endoscópico.

**Conclusão:**

A técnica cirúrgica descrita segue os requisitos básicos de cirurgia corretiva e possibilita boa visualização, avaliação e tratamento da placa atrésica e do terço posterior do septo, a fim de criar a nova abertura coanal. Pensamos que a utilização de um stent não é necessária, tal como recomendado no caso de outras técnicas cirúrgicas que envolvem o uso de mais retalhos de mucosas.

## Introduction

With a frequency of one in 5000–7000 births, choanal atresia (CA) is a rare congenital malformation of the nasal cavity characterized by the complete obliteration of the posterior choanae.^1^ CA was reported first by Roederer in 1755 while examining a newborn with total obstruction of the posterior nasal choana and later described by Otto in 1829 during an autopsy^2^, ^3^; the first surgical approach to CA was proposed in 1851 by Emmert, who first successfully corrected CA using transnasal surgery of the palate.^4^

Both genders are affected, with a male to female ratio of 1:2. In 70% of cases the malformation is mixed bony-membranous type, while in the remaining it is pure bony type.^5^ In the 67% of cases CA is unilateral, affecting mainly (71%) the right nasal cavity. In contrast to the unilateral form, which can be unrecognized for years, bilateral CA is a life-threatening condition often associated with dramatic clinical features like respiratory distress with feeding and intermittent cyanosis exacerbated by crying.

20%–50% of patients with CA, particularly those affected by bilateral forms, suffer also from other genetic malformations like CHARGE (choloboma, heart defects, CA, retardation of postnatal growth and mental development, genital hypoplasia and ear anomalies), Treacher Collins, Pfeiffer, Apert, Mandibulofacial dysostosis and Crouzon syndromes^6^; in such cases, because of the severe respiratory symptoms, a combined CA surgical treatment with tracheostomy is often necessary to guarantee safe airway management.

Hengerer and Strome attributed the CA embryological foundations to four factors: (1) Persistence of the buccopharyngeal membrane from the foregut; (2) Persistence of Hochstetter's bucconasal membrane; (3) Abnormal persistence or location of mesoderm in the choanal region; (4) Misdirection of the mesodermal flow, with an altered migration of neural crest cells which fail to reach their preordained position in the facial processes.^7^

Once CA has been diagnosed, surgical treatment remains the only therapeutic option. Several surgical approaches were previously reported, like transnasal, transantral, transpalatine and transeptal.

The aim of this paper was to report our experience in the surgical management of 18 cases of CA using a minimally invasive transnasal endoscopic approach with stentless single side-hinged flap technique.

## Methods

**Figure 1:**
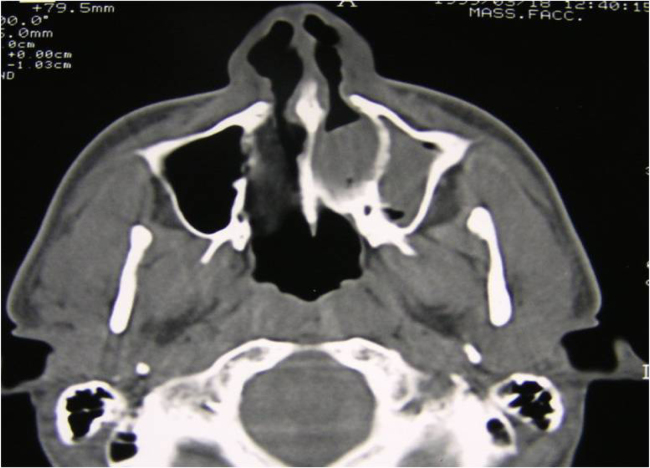
Axial CT scan, monolateral left bony choanal atresia.

**Figure 2:**
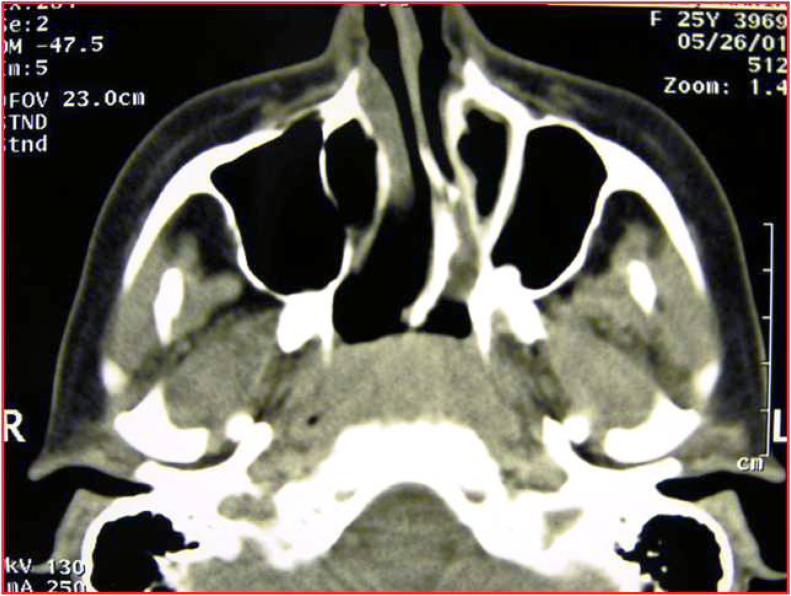
Axial CT scan, monolateral left mixed choanal atresia.

We evaluated the surgical results of 18 patients (ranging from 8–57 years of age) treated for CA between 2001 and 2005. Approval for this retrospective study was obtained from the local ethical committee (approval number V5605). Fourteen patients were affected by unilateral and bony CA (Fig. 1), one by unilateral and mixed (Fig. 2), one by bilateral and bony, and two by bilateral and mixed. Bilateral CA forms were reinterventions in patients who had previously undergone surgery at other departments. No cases of genetic syndromes were found. Clinical and radiological assessments were performed preoperatively in all patients and comprised a nasal endoscopy and a Maxillofacial CT scan to determine the extent of the atretic plate and to rule out any other craniofacial anomaly. The surgical procedure, under general anesthesia, was performed by 0° and 30° 4 or 2.7 mm telescopes (Karl Storz) depending on the age of the patient.

**Figure 3:**
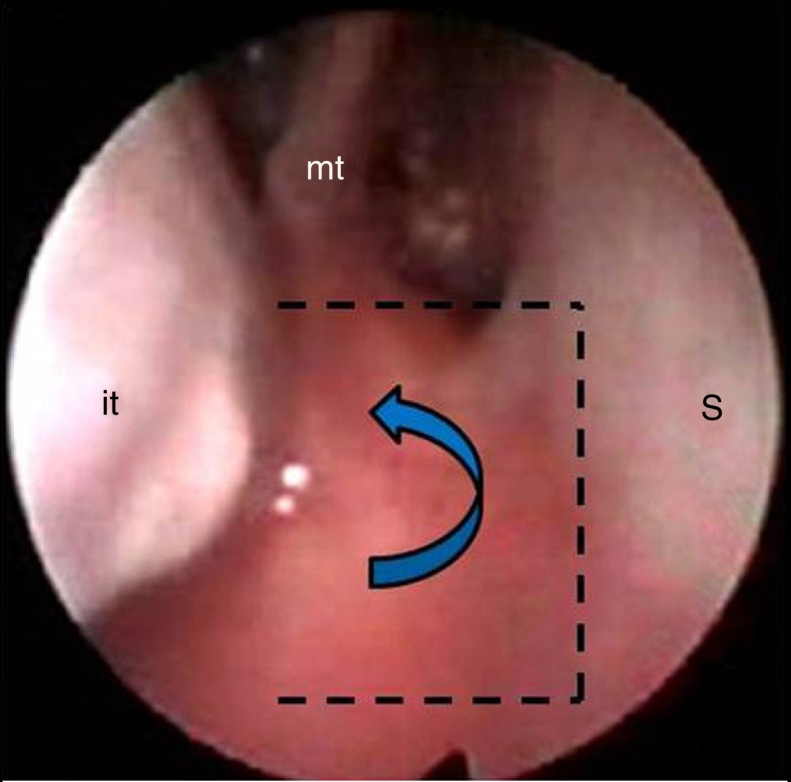
Endoscopic view: intranasal incisions on the atretic plate, fashioning a lateral flap (dotted lines). it, inferior turbinate; mt, middle turbinate; S, septum.

**Figure 4:**
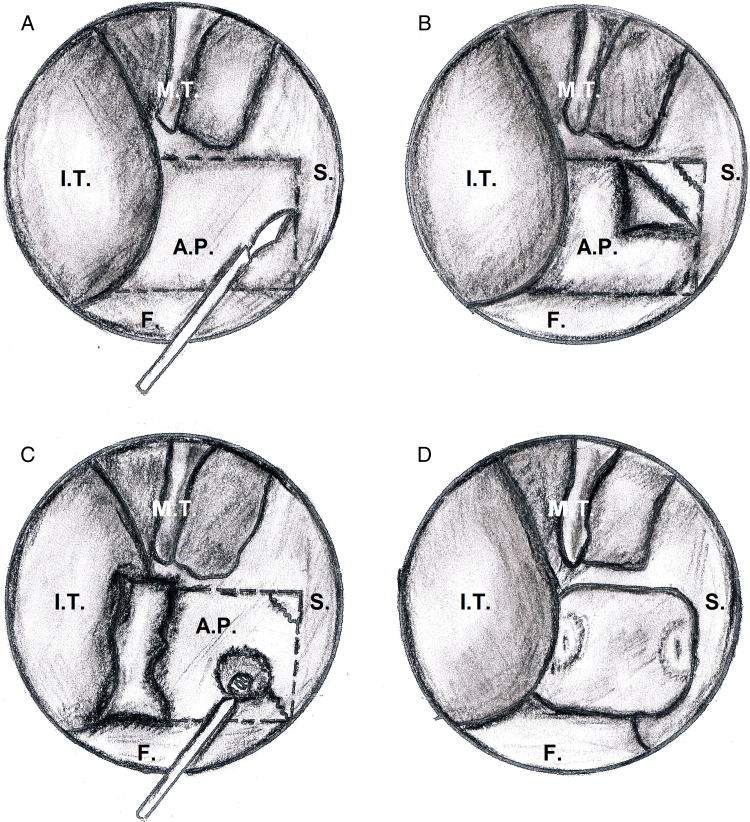
Main surgical steps of endoscopic choanoplasty technique from right nasal cavity: I.T., inferior turbinate; M.T., middle turbinate; S, septum, A.P., atretic plate; F, nasal floor.

We used a transnasal technique with a single side-hinged flap without stent placement. It is based on the following steps:

1. After the oral intubation, the nose is topically decongested. If nasal septum deviation exists, a hemitransfixion incision is created on the right of the nasal septum, with right subperichondral and bilateral subperiosteal dissection and correction of septal deformities performed.
2. Vertical incision of the nasal mucosa with a sickle knife at the junction of the atretic plate with vomer is created; by adding two horizontal incisions, one higher at the choanal arch and another lower at the edge between the atretic plate and the floor of the nasal cavity (Figure 3, Figure 4A), a side-hinged flap is elevated and laterally displaced (Fig. 4B and Fig. 5A). This step can be completed by the upward dislocation of the inferior turbinate in order to improve the exposure of the surgical field.

**Figure 5:**
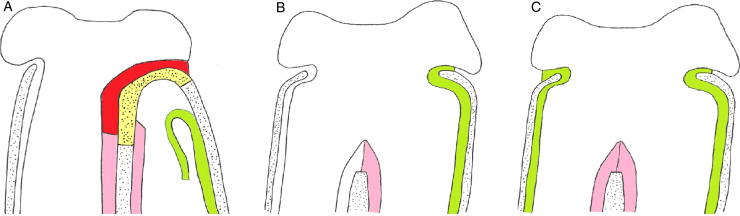
Laterally elevation of the mucoperiosteal flap and exposition of the atretic bony plate (A). Repositioning of the lateral mucosal flap covering the lateral nasal wall after removal of the atretic plate in case of monolateral (B) and bilateral atresia (C); atretic plate (yellow); mucosa of the posterior face of the atretic plate (red); mucosal flap laterally displaced (green); septal mucosa of each side (pink).
3. Perforation of the CA is performed at the level of its inferomedial portion and subsequent complete removal of the atretic plate together with the mucosa of the nasopharyngeal aspect with bone biting forceps or microdrill is carried out (Fig. 4C).
4. Resection of posterior third of the bony septum (vomer, ethmoidal lamina) with back-biting forceps and drill ensues, with bilateral vertical section with angled knife or micro-scissors of the septal mucosa so that only the posterior edge of the bony septum is resurfaced by approximation of the two mucoperiosteal septal sides (Figure 4, Figure 5). Finally, positioning of Merocel^®^ nasal packing from a minimum of 3 to a maximum of 7 days (mean 4.4) is favored. Stents were not placed in any case. No topical mitomycin or corticosteroids were applied.

In case of bilateral CA, the same procedure is performed on the contralateral side (Fig. 5C). During the immediate postoperative period an antibiotic therapy was administered and, after nasal packing removal, a nasal saline spray therapy at least twice a day for several weeks was recommended. Patients underwent a regular endoscopic follow-up to wash awasy crusts and secretions and verify choanal patency.

## Results

Table 1 shows clinical characteristics of the eighteen patients included in our study. Ten males and eight females (male/female ratio = 1:1.25), with a mean age at the time of surgery of 20.05 ± 11.32 years (median = 18.5 years), underwent CA treatment. Fifteen subjects (83.33%) had a bony atretic plate while three (26.77%) a mixed bony-membranous atretic plate. Two and sixteen cases suffered from bilateral and unilateral (9 left- and 7 right-sided) CA respectively. Two cases of bilateral CA were observed, an 8 year-old child and a 57 year-old woman, both affected by restenosis after initial surgical treatment at birth with simple perforation and stent placement. No patients suffered from Gastroesophageal Reflux Disease (GERD).

**Table 1:** Clinical characteristics of patients.

Cases	Gender	Age	Laterality	Type	Nasal packing	Restenosis
1	M	27 years	Right	Bony	4 days	No
2	M	25 years	Left	Bony	5 days	Yes
3	F	10 years	Right	Bony	4 days	No
4	M	20 years	Left	Mixed	3 days	Yes
5	F	9 years	Left	Bony	3 days	No
6	F	11 years	Left	Bony	3 days	No
7	M	13 years	Left	Bony	4 days	No
8	F	12 years	Right	Bony	4 days	No
9	M	8 years	Bilateral	Bony	7 days	No
10	F	57 years	Bilateral	Mixed	7 days	No
11	M	17 years	Right	Bony	4 days	No
12	M	27 years	Left	Bony	5 days	No
13	F	19 years	Left	Bony	4 days	No
14	M	21 years	Left	Mixed	4 days	No
15	M	15 years	Right	Bony	5 days	No
16	M	23 years	Left	Bony	3 days	No
17	F	29 years	Right	Bony	4 days	No
18	F	18 years	Right	Bony	5 days	No

All surgical procedures were completed within 140 min (surgical time range = 60–140 min; mean = 87 min). A septoplasty with a maxilla-premaxilla approach was performed in 7 patients (38.88%) who were affected by nasal septum deviation. CA treatment was not associated with adenoidectomy in the 8 and 9 year-old children (Patients 9 and 5). Hospitalization time ranged from 3 to 5 days (mean = 3.8 days). The mean time for nasal packing removal was 4.33 ± 1.18 days (median = 4 days). No intra- and/or early postoperative complications such as epistaxis, infection, erosion of the nares or intranasal synechiae occurred.

**Figure 6:**
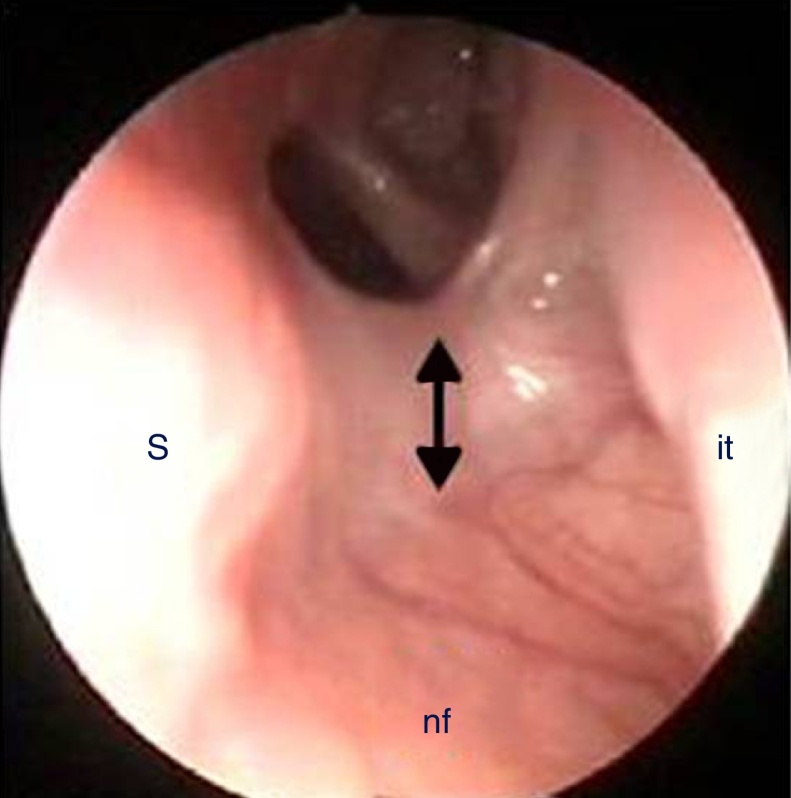
Partial restenosis on the nasal floor (arrow). S, septum; it, inferior turbinate; nf, nasal floor.

All patients underwent postoperative follow-up with nasal endoscopy. Overall follow-up period ranged from 1 to 10 years (mean 7.4 years). Between 2 and 3 months after surgical treatment two cases (11.11%) of partial restenosis (Patients 2 and 4) on the floor of the nasal cavity were found. Only one (5.55%) of these (Patient 4) presented a relapse of the nasal obstruction (Fig. 6) and was therefore successfully repaired with a second endoscopic procedure without positioning of a stent. The 16 remaining patients who underwent surgery had satisfactory functional patency of the choanae, without respiratory discomfort or secretions in the follow-up, and definite choanal patency was confirmed with nasal endoscopy.

## Discussion

Presently there is no unique or standardized technique in the management of CA. Surgical correction is closely related to patient age and anatomical characteristics of the CA itself: mono- or bilateral, partial or complete, membranous, bony or mixed.

Because of neonates are obligate nasal breathers, complete bilateral CA is a medical urgency which demands urgent temporary oral airway maintenance until adequate or clinically patent posterior nasal choanae is surgically established, generally with a trans-nasal approach.^8^ On the contrary, patients with unilateral or incomplete CA are often diagnosed and treated later in life, when patients seek medical attention because of long-standing unilateral nasal obstruction, anosmia and rhinorrhea.

Four main CA surgical approaches are described in the medical literature: trans-palatal, trans-antral, trans-septal, and trans-nasal. The trans-palatal approach offers a very wide field for operation, making corrective maneuvers easier, but is more invasive and susceptible to complications like bleeding, fistulas, infections and growth defects of the jaw and the palate bone.^9^, ^10^ The trans-antral approach is only of historical interest and also permits an adequate exposure of the surgical field, allowing a quick check for any bleeding and less risk of damaging the sphenopalatine arteries, veins and nerves, but can significantly increase the risk of deformities of growing structures such as the maxilla and upper teeth.^11^ The trans-septal approach is recommended in case of unilateral CA and in patients older than 8 years; it permits better correction of any deviations of the septum, resection of the posterior part of the vomer and preservation of mucosal flaps for coverage of the bleeding area.^12^

The trans-nasal approach is currently the most frequently used, due to the modern refinements of endoscopic techniques (it does not affect the growth of the mandibular arch, there are no malocclusions or cosmetic alterations to the face), especially in newborns with bilateral CA where a puncture of the atretic plate via the nostrils with subsequent use of Fearon dilators and stent placement are generally performed.^13^ In young patients, when the ethmoid sinuses have reached a satisfactory level of development, and in adults, it is possible to make incisions of the mucosa of the atretic plate, as reported by different authors. The most common techniques for incision are: double mucosal anterior and posterior low-hinged flap,^12^ side-hinged double flap,^14^, ^15^ upper hinged flap,^16^ four flaps with cruciate incisions,^11^, ^17^, ^18^ double nasal and septal flap,^19^ and multiple flaps secured with fibrin glue,^20^, ^21^, ^22^ so as to obtain mucosal flaps for the re-covering of the raw areas at the level of the medial lamina of the pterygoid process and the posterior part of the septum. Other authors instead did not flap techniques, such as El-Ahl et al. who performed a stentless transnasal endoscopic approach to treat bilateral CA in 7 neonates (ranging from 4 to 15 days of life) without evidence of restenosis.^23^ Additionally, to enlarge the choana to the maximum possible size, Liktor et al., suggested, in cases in which the atretic plate is suitably thin and the developmental status of the sphenoid sinus and the ethmoid cells is adequate, opening together the sphenoid sinus and the posterior ethmoid cells, resecting also the posterior pole of the middle turbinate; however, this modified technique may be considered to manage only selected cases like postoperative stenosis and unilateral CA in patients over 7 years of age.^24^

The topical application of mitomycin C, an aminoglycoside which inhibit fibroblast growth and migration, was also suggested to reduce risk of restenosis after surgery and improve the healing process; however its use is still controversial.^25^, ^26^, ^27^, ^28^, ^29^, ^30^ For example, Bozkurt et al., studying 12 patients who underwent surgery for choanal atresia with and without the use of mitomycin C, reported no case of restenosis in the first group and formation of granulation tissue in the 42.9% of the second group.^30^ On the contrary, Uzomefuna et al. did not find any significant difference between patients who were treated at initial surgery with topical mitomycin C and patients who had no mitomycin C application (53% vs. 60%).^31^

Successful CA surgical outcomes are influenced by the presence/absence of factors like nasopharyngeal reflux, GERD, age <10 days (associated with limited visualization in noses of neonates and limited resection of the vomer), bilateral CA with purely bony atretic plate and associated malformations.^9^, ^27^ None of these risk factors was identified in our patients with the exception of bilateral CA, found in two subjects (11.11%) who experienced no restenosis after our treatment.

Our study, with only two cases (11.11%) of partial restenosis, showed good surgical outcomes without the use of a postoperative stenting. Similar rate of restenosis (14%) were reported by Ibrahim et al. who performed also an endoscopic stentless choanoplasty with a single side mucoperiosteal flap to treat 21 CA children.^32^ It is difficult to make a real comparison because of the different demographic characteristics of the sample studied, the high number of bilateral CA included (11/21) and the shorter follow-up period.

It is clear that a stentless technique avoids the potential stent-related complications (such as discomfort, localized infection and ulceration, circumferential scar or granulation tissue formation) but needs to be associated with a close post-operative follow-up.^33^ However, as reported in a systematic review by Cedin et al., with a absolute risk for not needing a reoperation (0.81), the comparison between surgeries with and without stent did not prove any significant evidence in favor of a specific technique.^34^

## Conclusion

The surgical CA approach described is technically easy to perform and allows good visualization, evaluation and treatment of the atretic plate and the posterior third of the septum. Our data, with only two cases of partial restenosis (11.11%) show good surgical outcomes without the use of stenting. However, due to the number of patients included, these findings cannot be generalized and a larger sample is necessary to obtain statistically significant conclusions. We suggest the use of this transnasal endoscopic surgery because it follows the basic requirements of a minimally-invasive corrective approach: the creation of a widely patent posterior nasal choana sufficient for normal bilateral nasal breathing, absence of secretion accumulation, minimization of endonasal scar tissue formation and prevention of abnormal craniofacial growth in children who have not reached their full growth yet.

## Conflicts of interest

The authors declare no conflicts of interest.
